# The BioGRID database: A comprehensive biomedical resource of curated protein, genetic, and chemical interactions

**DOI:** 10.1002/pro.3978

**Published:** 2020-11-23

**Authors:** Rose Oughtred, Jennifer Rust, Christie Chang, Bobby‐Joe Breitkreutz, Chris Stark, Andrew Willems, Lorrie Boucher, Genie Leung, Nadine Kolas, Frederick Zhang, Sonam Dolma, Jasmin Coulombe‐Huntington, Andrew Chatr‐aryamontri, Kara Dolinski, Mike Tyers

**Affiliations:** ^1^ Lewis‐Sigler Institute for Integrative Genomics Princeton University Princeton New Jersey USA; ^2^ The Lunenfeld‐Tanenbaum Research Institute Mount Sinai Hospital Toronto Ontario Canada; ^3^ Arthur and Sonia Labatt Brain Tumor Research Center and Developmental and Stem Cell Biology The Hospital for Sick Children Toronto Ontario Canada; ^4^ Institute for Research in Immunology and Cancer Université de Montréal Quebec Canada

**Keywords:** biological network, chemical interaction, COVID‐19, CRISPR screen, drug target, genetic interaction, phenotype, post‐translational modification, protein interaction, ubiquitin‐proteasome system

## Abstract

The BioGRID (Biological General Repository for Interaction Datasets, thebiogrid.org) is an open‐access database resource that houses manually curated protein and genetic interactions from multiple species including yeast, worm, fly, mouse, and human. The ~1.93 million curated interactions in BioGRID can be used to build complex networks to facilitate biomedical discoveries, particularly as related to human health and disease. All BioGRID content is curated from primary experimental evidence in the biomedical literature, and includes both focused low‐throughput studies and large high‐throughput datasets. BioGRID also captures protein post‐translational modifications and protein or gene interactions with bioactive small molecules including many known drugs. A built‐in network visualization tool combines all annotations and allows users to generate network graphs of protein, genetic and chemical interactions. In addition to general curation across species, BioGRID undertakes themed curation projects in specific aspects of cellular regulation, for example the ubiquitin‐proteasome system, as well as specific disease areas, such as for the SARS‐CoV‐2 virus that causes COVID‐19 severe acute respiratory syndrome. A recent extension of BioGRID, named the Open Repository of CRISPR Screens (ORCS, orcs.thebiogrid.org), captures single mutant phenotypes and genetic interactions from published high throughput genome‐wide CRISPR/Cas9‐based genetic screens. BioGRID‐ORCS contains datasets for over 1,042 CRISPR screens carried out to date in human, mouse and fly cell lines. The biomedical research community can freely access all BioGRID data through the web interface, standardized file downloads, or via model organism databases and partner meta‐databases.

## 1 INTRODUCTION

The biomedical literature contains a vast amount of information that captures a great deal of knowledge about biology and biomedicine. However, identifying relevant information about gene or protein function with respect to any given process or disease can be a monumental task because of the sheer volume of data contained in the scientific literature and the fact that much of the literature is not open access. Automated extraction of key data elements from publications cannot be easily achieved due to the unstructured free‐form text that comprises most of the biomedical literature. Moreover, relevant data are often only present in nontext elements such as figures, tables, and supplementary information. A fundamental goal of biomedical data curation is to convert text‐, table‐, and figure‐based experimental information from the literature into consistent structured records that can be easily accessed in standardized formats for computational analyses.

**FIGURE 1:**
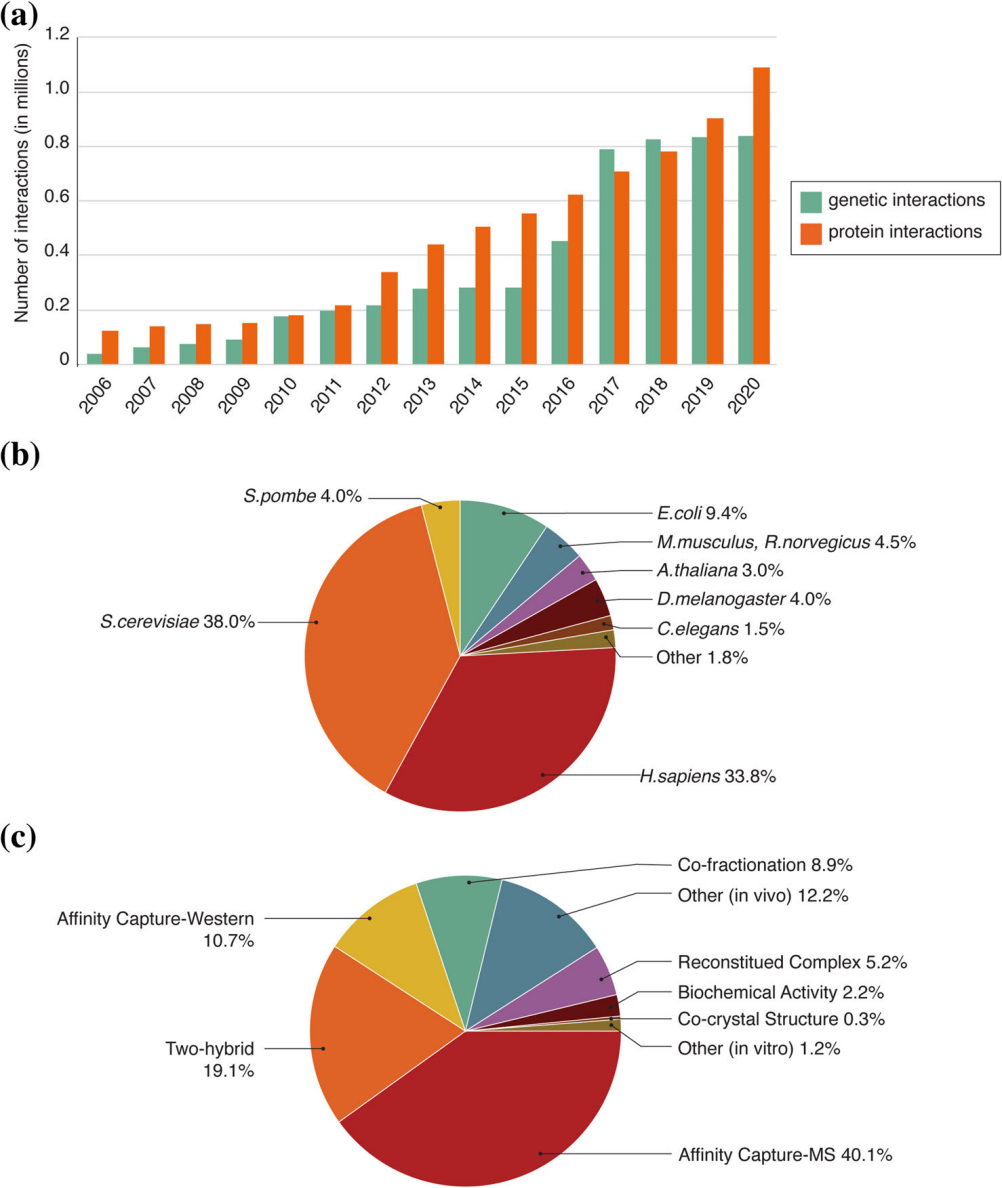
Summary of biological interactions in BioGRID. (a) Annual increase of protein and genetic interaction records in BioGRID. (b) Percentage of interactions from human, yeast, mouse/rat, worm, fly, and other species in BioGRID release 4.1.190. (c) Protein interactions as a function of experimental evidence codes. Main evidence codes are indicated, minor evidence codes are grouped as “other.” Detailed description of evidence codes can be found at https://wiki.thebiogrid.org/doku.php/experimental_systems

To address these issues, the BioGRID resource was started in 2006 with the focused goal of comprehensively curating all available biological interaction data generated in the budding yeast *Saccharomyces cerevisiae*.^1^, ^2^ Discrete interactions between macromolecules and/or functional genetic elements form the basis for all biological systems and collectively form highly interconnected auto‐regulated networks that imbue system‐level properties on cells, tissues, and organisms. These discrete types are readily exploited in computational approaches to model network behavior.^3^ Budding yeast represented an ideal test case for curation of interaction data because of the implementation of high‐throughput genetic and proteomic techniques that enabled the first genome‐wide analyses of genetic and protein interactions in any species.^4^ Since then, the BioGRID has expanded coverage to include interaction data for all major model organisms and humans, as well as many other less well‐studied species, more than 70 species in all. As of October 2020, BioGRID contains over 1.93 million protein and genetic interactions curated from more than 63,000 publications (Figure 1a, Table 1). With respect to specific species, the BioGRID currently contains over 755,000 interactions for budding yeast, 79,000 interactions for fission yeast, 670,000 interactions for human, 29,000 interactions for worm, 78,000 interactions for fly, and 300,000 interactions for all other organisms (Figure 1b). BioGRID has also expanded its curation strategy to include other types of data that are relevant to biological interactions. For example, BioGRID records more than 515,000 unique protein post‐translational modifications (PTMs) and over 28,000 interactions between drugs or other chemicals and their protein targets. BioGRID also now curates gene‐phenotype relationships from genome‐wide CRISPR screens. The BioGRID record structure, database architecture, and curation pipeline have been described in detail elsewhere.^5^

**TABLE 1:** Interaction datasets for main species in BioGRID

Species	Type	Gene or protein nodes	Interactions (redundant)	Interactions (nonredundant)	Publications
*S. cerevisiae*	P	7,077	175,968	117,040	9,699
	G	5,962	579,323	443,268	9,392
	P + G	7,332	755,291	546,582	16,037
*S. pombe*	P	3,571	17,529	13,109	1,624
	G	3,627	62,313	52,449	2,003
	P + G	4,651	79,842	64,418	2,874
*A. thaliana*	P	10,737	58,424	50,756	2,307
	G	306	351	283	155
	P + G	10,787	58,775	50,952	2,387
*C. elegans*	P	6,804	27,225	26,119	207
	G	1,136	2,344	2,277	36
	P + G	7,164	29,569	28,353	226
*D. melanogaster*	P	9,226	64,404	54,507	3,697
	G	3,147	14,476	10,141	4,395
	P + G	9,546	78,880	63,290	7,166
*M. musculus*	P	15,990	80,510	72,965	4,213
	G	355	394	349	198
	P + G	16,039	80,904	73,236	4,351
*H. sapiens*	P	25,722	663,179	506,159	32,168
	G	3,767	9,181	9,055	351
	P + G	26,126	672,360	514,501	32,310
Other	P	22,634	57,877	49,945	3,279
	G	4,536	171,910	170,197	118
	P + G	25,045	229,787	219,323	3,359
All	P	76,711	1,088,662	841,748	51,784
	G	22,191	839,711	687,467	16,448
	P + G	81,183	1,928,373	1,511,287	63,083

*Note*: Data are compiled from BioGRID release 4.1.190 of October 1, 2020.

Abbreviations: G, genetic interaction; P, physical interaction.

Due to the vast extent of the human biomedical literature, BioGRID has in part taken a biological process‐ and/or disease‐focused approach in order to build curation depth in critical areas of human biology. These themed curation projects include the ubiquitin proteasome system (UPS), chromatin modification, autophagy, glioblastoma, Fanconi anemia and, most recently the SARS‐CoV‐2 coronavirus that is the causative agent of the COVID‐19 pandemic.^6^ A curated gene/protein list is developed by domain experts for each project to guide the literature curation strategy. These focused projects have driven much of the extensive growth of the human interaction curation in BioGRID over the past 10 years and are discussed in detail below. The themed curation efforts are complemented by a dedicated on‐going effort to curate all large‐scale human interaction datasets.

In this review, we describe the content and functionality of BioGRID, with an emphasis on the newest developments. We highlight recent interaction curation for the UPS and for the emergent coronaviruses that cause severe acute respiratory syndromes, including SARS‐CoV‐2. We also describe a new extension of BioGRID, named the Open Repository of CRISPR Screens (ORCS), which currently contains over 1,042 annotated CRISPR phenotype screens. The large collection of curated interaction data in BioGRID represents a unified resource for integrative network analyses by computational biologists and enables efficient mining of the biomedical literature by researchers interested in specific genes or proteins.

## 2 PROTEIN AND GENETIC INTERACTIONS

All protein and genetic interactions annotated in BioGRID are exclusively derived from expert manual curation of experimental data reported in peer‐reviewed publications. Each experimental result that supports an interaction is assigned by curators to a structured experimental evidence code with an accompanying PubMed identifier. BioGRID uses custom structured vocabularies to describe interaction data, which includes 17 different protein interaction evidence codes (e.g., affinity capture‐mass spectrometry, co‐crystal structure, Förster resonance energy transfer [FRET], and two‐hybrid) and 11 genetic interaction evidence codes (e.g., synthetic lethality, synthetic rescue, and dosage growth defect). Detailed curation guidelines for each evidence code method are explained in the BioGRID help wiki (wiki.thebiogrid.org). High throughput interaction datasets are typically extracted from online supplementary files and converted by curators into a consistent format for upload into BioGRID. The breakdown of protein interactions in BioGRID by the most commonly used experimental methods is shown in Figure 1c, 90% of which derive from in vivo experimental methods. Experimentally unsupported statements in the literature and predicted interactions based on computational methods are not included in order to maintain BioGRID as a high‐confidence interaction database of primary experimental results. For this reason, BioGRID interaction data have frequently been used as a gold standard in computational studies, either as training sets for machine learning or for verification of predicted results.^3^, ^7^, ^8^

BioGRID houses interactions from all major model organisms (Figure 1b, Table 1). We maintain complete coverage of both low‐ and high‐throughput protein and genetic interaction data from *S.cerevisiae* and *S.pombe*, which makes BioGRID the most comprehensive database available for yeast researchers to explore and analyze protein and gene interaction networks. While it is not feasible to curate the many millions of low throughput studies reported in PubMed for more complex model organisms and humans, BioGRID nevertheless aims to capture all large‐scale studies for widely‐used model systems. BioGRID partners with model organism databases (MODs) including SGD,^9^ PomBase,^10^ FlyBase,^11^ Wormbase,^12^ and the Bio‐Analytic Resource for Plant Biology (BAR).^13^ These important partnerships allow sharing of the extensive curation load and wide dissemination of interaction data to specific research communities. BioGRID interactions are integrated within MODs, and users are able to navigate between these sites and BioGRID via reciprocal links.

**FIGURE 2:**
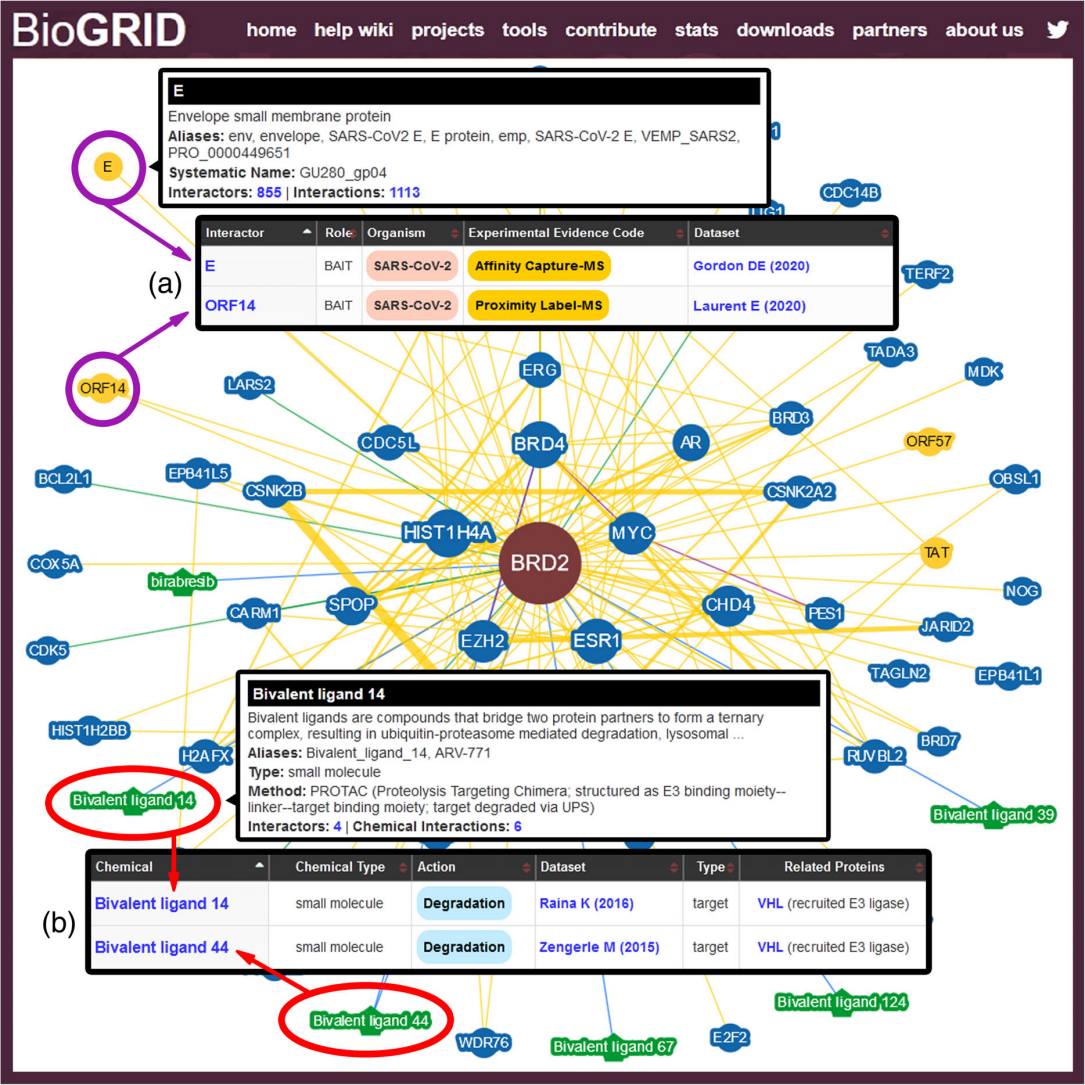
BioGRID result summary page (a) Network viewer provides a graphical representation of the protein, genetic and chemical interactions for any protein/gene of interest. Results can be filtered for interaction type and evidence threshold. Heterologous interactions between two different species are indicated in yellow, as shown here for the interaction between human BRD2 and SARS‐CoV‐2 proteins (purple circles). (b) Chemical interaction nodes are shown in green and can also be viewed as a list by selecting the Chemical Interaction view in the Switch View bar, as shown here for Bivalent ligand 14 and Bivalent ligand 44 (red circles)

Several parallel routes enable full access to BioGRID content. For computational biologists interested in a large number of curated interactions, the downloadable file repository holds formatted data files of the most current release as well as archived monthly releases, which are important for future validation or refinement of analytical methods. Downloads can be customized as needed to create complex interaction datasets that may be visualized using tools such as Cytoscape (cytoscape.org),^14^ NDEx (www.ndexbio.org)^15^ or EsyN (www.esyn.org),^16^ and also used for various analytical methods. Importantly, the BioGRID REST (webservice.thebiogrid.org) and BioGRID ORCS REST (orcsws.thebiogrid.org) services provide an open, standardized application programming interface (API) for retrieval of all curated data. These REST services currently allow more than 1,000 different software applications and pipelines to automatically import BioGRID data records. Automated retrieval of BioGRID data is also available through the widely‐used PSICQUIC interface.^17^ Individual users interested in interactions for specific genes or proteins can use the search interface at the top of the BioGRID main page. The BioGRID search interface allows users to rapidly query a gene, protein, or chemical of interest for reported interactions, and displays all experimental methods used to detect the interaction as well as the specific publications that report the data. Searchable identifiers include gene/protein names, known aliases, systematic open reading frame (ORF) names, chemical names or formulas, and other resource identifiers. For example, a search on the term BRD2 returns a list of all genes/proteins that contain BRD2 as an identifier, as ranked by number of associated BioGRID interactions. Selecting the human entry for BRD2 brings the user to the Result Summary page, which lists all reported interactors for human BRD2. Multiple viewing options are available from the Switch View toolbar, which allows users to generate a graphical network representation of the data that includes curated chemical, protein, and genetic interactions (Figure 2a). When available, PTM sites are displayed in a separate protein sequence viewer. Another available search option is to search by publication using either PubMed ID or keywords, which will return either a list of papers or a unique Publication Summary page. Each paper annotated by curators has a Publication Summary page that outlines all the interactions from that publication.

## 3 PROTEIN PTMs

Regulated protein interactions governed by PTMs underpin virtually all cellular responses to developmental cues, environmental signals, and stress.^18^ BioGRID actively curates a number of predominant PTMs including protein phosphorylation, the conjugation of ubiquitin and its related small protein modifiers such as NEDD8 and SUMO, and other small chemical modifications. The BioGRID currently contains more than 458,000 site‐specific nonredundant protein PTMs curated from over 4,600 publications for yeast, mouse, human, and other species. While a fraction of these PTM data are curated from low throughput studies, most are derived from high‐throughput mass spectrometry studies that are able to identify thousands of modification sites for any given PTM type in a single experiment.^19^ An additional 57,000 documented PTMs that have not been assigned specific sites have also been curated. BioGRID users can access protein modification sites in the PTM view on protein summary pages, which display all available PTM information and the corresponding amino acid modification site within the protein sequence. All PTM data are made available in a standardized format as bulk download files.

Protein kinases and phosphatases have evolved to control cellular responses to myriad different signals, such that protein phosphorylation is the predominant reversible protein modification.^20^ Curated phosphorylation sites for budding yeast total nearly 20,000 phosphosites for over 3,100 proteins in BioGRID. As for other PTMs, these protein phosphosites are also indicated as a function of residue position in the PTM view on protein summary pages. Enzyme‐substrate interactions are also displayed on the Yeast Kinome Project page (thebiogrid.org/project/2), which classifies interaction partners as kinases, phosphatases or accessory subunits.^21^, ^22^ Often these site assignments are condition‐specific and/or supported by site‐specific mutagenesis data.

In another example of particular interest, BioGRID has annotated approximately 383,700 nonredundant ubiquitin modification sites mapped on more than 54,900 human proteins, as well as 40,000 unique sites mapped to more than 3,900 budding yeast proteins. E3‐substrate relationships that have been demonstrated with in vitro enzymology evidence are also captured as protein interactions in BioGRID using the evidence code biochemical activity with ubiquitination noted as the modification. Within the UPS themed project, sites of ubiquitin modification identify ubiquitinated substrates in particular contexts and can help to establish substrate‐E3 relationships.

## 4 THEMED CURATION

As currently more than 19 million publications are retrieved from PubMed with the search term “human,” curation of the entire corpus of literature that contains potential human gene/protein interactions is a wholly impractical task even when narrowed by additional search terms. To partly circumvent this issue, BioGRID has taken a focused project‐based approach to curation of human protein interactions, allowing for curation of manageable collections of high impact data. BioGRID themed projects represent central biological processes with disease relevance such as chromatin modification, autophagy and the UPS, or diseases of interest including glioblastoma, Fanconi Anemia and COVID‐19. These projects begin with the generation of a core gene/protein list by curators, which entails literature review for evidence of protein function, consultation with domain experts, and inspection of relevant annotated functional domains and GO pathways. These gene lists are then used to identify papers containing relevant biological interactions, which are captured in BioGRID using the appropriate interaction evidence codes (Table 2). As simple keyword searches recover a preponderance of publications that do not contain interaction data,^23^ themed searches often generate queues of candidate papers that still present a formidable challenge for both initial and maintenance curation. To narrow curation queues to the most salient publications, text‐mining tools such as PieTheSearch^24^ are used to prioritize papers for curation in larger projects. To date, BioGRID curators have read more than 197,000 publications selected by either keyword searches or text‐mining approaches and of these more than 73,000 contain direct experimental evidence for biological interactions and/or PTM data. Importantly, themed curation projects are kept current through regular updates. BioGRID continues to add themed projects in response to the needs of the research community, as illustrated by the recent addition of a COVID‐19 Coronavirus Project, discussed in more detail below.

**TABLE 2:** BioGRID themed curation projects

Project	Genes	Papers	Interactions
Autophagy	142	2,099	16,848
Coronavirus	110	204	20,352
Chromatin Remodeling	470	11,609	101,800
Fanconi Anemia	20	1,072	6,013
Glioblastoma	35	3,545	13,464
UPS‐Human	1,285	15,401	165,883
UPS‐Yeast	299	3,630	100,271

*Note*: Data are compiled from BioGRID release 4.1.190 of October 1, 2020.

A significant sustained curation effort underpins the current largest themed project at BioGRID, namely curation of the protein, genetic, and chemical interactions of the UPS. The conjugation of ubiquitin to substrate proteins controls the stability, localization, and/or function of much if not most of the proteome.^25^ The UPS project is based on an exhaustive and continuously updated list of 1,285 human genes and 299 budding yeast genes that encode the E1, E2, and E3 enzymes that catalyze ubiquitin conjugation to substrates, the deubiquitinating enzymes that remove ubiquitin, the ubiquitin binding domain proteins that read the ubiquitin code, and subunits of the 26S proteasome that mediate the proteolytic destruction of many ubiquitinated proteins. To date, BioGRID has curated over 266,100 UPS protein interactions from more than 19,000 publications. In addition, BioGRID curators have captured sites of ubiquitin modification, largely from high‐throughput mass spectrometry‐based studies, and chemical modulators of UPS function, including a novel and promising class of prospective therapeutic drugs designed to re‐wire the UPS, which are described as an example of chemical interaction curation below.

## 5 SARS‐COV‐2 AND OTHER CORONAVIRUS PATHOGENS

The emergence of the Coronavirus Disease 2019 (COVID‐19) pandemic in early 2020 led to a critical need to identify new therapeutic treatment options. To assist in this urgent drug discovery effort, BioGRID pivoted curation toward the capture of coronavirus‐related interaction data to help identify potential drug targets and lead compounds for drug repurposing. In March 2020, BioGRID began curation of interactions related to Severe Acute Respiratory Syndrome Coronavirus 2 (SARS‐CoV2), the causal agent of COVID‐19.^26^ SARS‐CoV‐2 is a betacoronavirus related to SARS‐CoV and MERS‐CoV, two other zoonotic viruses that caused Severe Acute Respiratory Syndrome (SARS) in 2002 and the Middle East Respiratory Syndrome (MERS) in 2012, respectively.^27^ To allow curation, coronavirus protein identifiers for SARS‐CoV‐2, SARS‐CoV and MERS‐CoV were added to BioGRID. The genomes of these three coronavirus are similar,^28^, ^29^ and the encoded protein differences are accounted for in BioGRID annotation. The SARS‐CoV‐2 genome has two large ORFS, Orf1a and Orf1ab, at the 5′ end that encode polyproteins that are proteolytically cleaved into 16 nonstructural proteins (nsp1–nsp16) by two virus‐encoded proteases, the main protease Mpro (3CLpro) and the papain‐like protease PLpro.^30^ The 3′ end of the viral genome encodes the remaining 13 ORFs including 9 accessory factors and four structural proteins: Spike (S), Membrane (M), Envelope (E), and Nucleocapsid (N). The genome of SARS‐CoV is similar except for the 3′ open reading frame: SARS‐CoV encodes Orf8a and Orf8b whereas Orf8 of SARS‐CoV‐2 is intact. MERS‐CoV, on the other hand, has a slightly different genomic arrangement of the E, M, and N genes and unlike the other two SARS‐related viruses, encodes only 5 accessory factors.^28^ Viral‐human protein interactions for these coronaviruses were extracted from primary publications as well as associated supplementary data files and made available through the BioGRID web interface and in customized download files. As many relevant studies on SARS‐Cov‐2 have been released as preliminary prereports in bioRxiv (www.biorxiv.org) and medRxiv (www.medrxiv.org) prior to peer‐reviewed publication, BioGRID has altered its curation protocols to allow data from preliminary reports. Once preprint data is published in a reviewed journal, the data is re‐curated and replaces the preprint data in BioGRID. To expedite curation of newly identified SARS‐CoV‐2 host protein interactions, an in‐house text analyzer developed by BioGRID has enabled rapid triaging of preprints from nonpeer reviewed sources. Expansion of this project to other related coronaviruses of global health interest, SARS‐CoV and MERS, also allowed for the curation of relevant interactions with viral protein orthologs. As of October 2020, BioGRID has reviewed over 1,300 published papers and preliminary reports resulting in the capture of over 20,000 coronavirus interactions. These interactions are displayed on the relevant BioGRID protein summary pages and through a new COVID‐19 Coronavirus Project page (thebiogrid.org/project/3/covid-19-coronavirus-project.html). For example, interactions of the BRD2 chromatin reader with the SARS‐CoV‐2 Envelope (E) protein and ORF14 protein^30^, ^31^ are shown in the network view for BRD2 (Figure 2a). This interaction, coupled with the fact that BRD2 is known to regulate the transcription of over 1,400 genes,^32^ suggests that it may be worth exploring BRD2 as a potential host cell target to mitigate COVID‐19 symptoms.^30^ Known inhibitors of many other host cell factors that interact with viral proteins^30^, ^31^, ^33^ or host cell signaling networks altered by viral infection^34^ represent candidates for repurposed therapeutics against SARS‐CoV‐2.

## 6 CHEMICAL‐PROTEIN INTERACTIONS

BioGRID is one of the few resources that combines manually curated chemical‐protein interaction data with relevant protein and genetic interactions. Database integration of chemical‐target and biomolecular interaction networks can help guide drug repurposing, which is faster and more cost‐effective compared to de novo drug development.^35^ For most drugs that have been repurposed, new indications have been discovered through serendipity. A more rational approach to repurposing may instead begin at the drug, target, or local network level.^35^ Protein interaction networks generated from BioGRID data can facilitate each of these approaches.

To facilitate network‐based approaches to drug discovery, BioGRID has integrated manually curated chemical–protein interactions to allow for the display of small molecule‐target data in relation to protein and genetic interactions. BioGRID chemical interaction records use a minimal unified set of fields that allow interoperability between multiple resources, as well as a standard downloadable format for computational analyses. The minimal records for each small molecule interaction are displayed in a chemical view available on the Result Summary page for each protein or gene. The minimal record structure has been designed to allow for efficient import of data into BioGRID from various other drug interaction resources, including DrugBank (www.drugbank.ca),^36^ ChemSpider (www.chemspider.com), BindingDB (www.bindingdb.org),^37^ and the PDB (www.wwPDB.org).^38^ The original sources for each chemical record are cited with links to each database, allowing users to directly access the original data source for additional details. The BioGRID network viewer overlays these chemical‐target interactions onto their corresponding protein and genetic interaction networks, enabling researchers to visually connect a bioactive compound of interest to a relevant drug target along with its local interaction network.

In a themed chemical curation project that complements the comprehensive curation of human UPS interactions, BioGRID curators have systematically annotated bioactive compounds that inhibit or activate human UPS enzymes. These bioactive compounds target each class of UPS enzyme including the E1, E2, and E3 enzymes that mediate substrate ubiquitination, deubiquitinating enzymes, and the 26S proteasome. In addition to conventional small molecule modulators of UPS enzymes, BioGRID has curated a novel class of compounds referred to as bivalent ligands (BVLs) that have been engineered to bridge substrates and E3 ubiquitin ligases and thereby induce the targeted degradation of specific proteins of therapeutic interest. These bifunctional small molecules include PROTACs (protein‐targeting chimeric molecules), HaloPROTACs, and SNIPERs (specific and nongenetic IAP‐dependent protein erasers), as well as molecular glue compounds such as the IMiDs (immunomodulatory imide drugs).^39^, ^40^ The BVL concept has recently been expanded to exploit additional degradation mechanisms including for lysosome‐targeting chimeras (LYTACs)^41^ and autophagy‐targeting chimeras (AUTACs, also termed ATTECs).^42^, ^43^ To accommodate developments in this rapidly advancing technology area, a dedicated new record structure has been implemented to capture the attributes of each bivalent molecule and assign a unique BVL designation (i.e., BVL #) in order to unify complex names that are reported in the literature. This BVL designation allows for a simple display when visualized in a network graph, while the original published names for BVLs are provided in associated notes for ease of disambiguation. The BVL record structure also includes a method field that indicates the type of BVL and a brief description of how the BVL achieves target protein degradation, inhibition or other functional alteration. Results for BVLs are displayed in the chemical view for the recruited E3 ligase and target protein. As an example, BVL 14 uses linked chemical moieties to recruit the E3 ligase VHL to initiate degradation of the target protein BRD2 (Figure 2b). To date, chemical‐protein interaction data has been compiled for 168 unique BVLs as well as 149 UPS inhibitors. To our knowledge, BioGRID is the only unified resource containing UPS protein‐chemical interactions for conventional small molecule inhibitors along with novel bifunctional compounds that mediate targeted protein degradation.

As of October 2020, the entire chemical‐protein interaction dataset in BioGRID, including the unique collection of UPS inhibitors and BVLs, comprises over 28,000 chemical‐protein interactions involving approximately 5,300 bioactive compounds curated from more than 9,300 publications (Table 3). The vast majority of these records document small molecule‐target interactions but a subset represent approximately 165 biologics that include protein‐based therapeutics such as insulin^44^ and antibody‐based therapeutics such as the immune checkpoint inhibitor nivolumab.^45^ BioGRID will focus future chemical interaction curation on small molecules and biologics that target SARS‐CoV‐2 and related coronavirus proteins in order to facilitate network‐based drug repurposing and de novo drug discovery against COVID‐19.^30^, ^34^, ^46^ All chemical‐protein interactions can be found on the relevant protein search result pages and rendered in the on‐line network viewer, and can be downloaded in standardized formats for further analysis.

**TABLE 3:** BioGRID chemical interaction datasets

Organism	Interactions (redundant)	Interactions (nonredundant)	Unique genes	Unique publications	Unique chemicals
*H. sapiens*	26,127	11,040	2,206	9,084	4,759
*E. coli*	1,590	834	287	137	583
*C. albicans*	108	25	7	50	24
HIV1^a^	105	53	2	48	53
Other^b^	449	249	93	100	187
Total	28,379	12,201	2,595	9,359	5,339

*Note*: Data are compiled from BioGRID release 4.1.190 of October 1, 2020.

^a^ Human Immunodeficiency Virus 1.

^b^ Other mammalian, yeast, bacteria, or viral species.

## 7 CURATION OF GENOME‐WIDE CRISPR SCREENS FOR GENE‐PHENOTYPE RELATIONSHIPS

Protein function can be assigned by biochemical, cell biological, bioinformatics and genetic methods, each of which affords a complementary perspective. In particular, cellular or organismal phenotypes caused by loss or gain of function alleles represent one of the primary means of understanding protein function. Moreover, genetic interactions between two or more alleles that further alter phenotypes can be used to infer protein interactions.^47^ A substantial fraction of known human proteins remain poorly characterized, due in large part to a dearth of definitive genetic methods to assign function. Indeed, the function of most human proteins was first inferred from studies of orthologs in genetically tractable model organisms. Although RNAi‐based screening technologies have to some extent helped with human protein annotation, these methods have been fraught with nonspecific off‐target effects. This situation has changed dramatically with the discovery of the clustered regularly interspaced short palindromic repeats (CRISPR) genome editing system in bacteria and the subsequent development of CRISPR‐based screening technologies.^48^

Programmable CRISPR‐based endonucleases in conjunction with genome‐wide pools of single guide RNAs (sgRNAs) that direct the nuclease to specific sites in the genome have revolutionized genetic screens in human cell lines and other animal model systems.^48^ These RNA‐guided CRISPR nucleases, such as the original and widely‐used Cas9 enzyme, generate a precise cut at each targeted site, which results in high frequency indel formation and frequent loss‐of function for each protein‐coding sequence. Cas9 has also been fused to various regulatory domains to allow transcriptional activation and repression screens.^49^ Other CRISPR nucleases with advantageous properties, such as Cas12a, Cas9‐Cas12a hybrids, the Cas13 RNA nuclease, and a recently described compact enzyme called Casϕ have been developed for similar applications.^50^, ^51^, ^52^ CRISPR‐based genetic screens have now been reported in numerous publications that link gene function to cell viability, chemical and stress resistance, and other phenotypes.

To increase the accessibility of CRISPR screen data and facilitate assignment of protein function, BioGRID has recently developed an embedded resource called the Open Repository of CRISPR Screens (ORCS, orcs.thebiogrid.org) to house and distribute comprehensive collections of CRISPR screen datasets curated from the literature. Given that CRISPR nuclease formats, sgRNA libraries, experimental methods and scoring algorithms vary substantially from one publication to another, BioGRID curators use a custom record structure called MIACS (minimal information about CRISPR screens) to capture salient screen parameters and metadata.^53^ BioGRID‐ORCS curation is updated on a regular basis and currently includes 1,042 CRISPR screens from 114 distinct publications representing more than 60,000 unique genes across three species (human, mouse and fly) in over 670 cell lines (Table 4). ORCS also curates datasets in preliminary preprint reports and/or provided directly by authors in advance of publication in the primary literature.

**TABLE 4:** Summary of BioGRID‐ORCS datasets

Organism	Screens	Genes	Publications	Cell lines	Cell types
*D. melanogaster*	3	13,615	1	1	1
*H. sapiens*	1,008	22,823	100	656	105
*M. musculus*	31	23,871	15	14	10
All	1,042	60,309	114	671	112

*Note*: Data are compiled from BioGRID ORCS release 1.1.6 of October 1, 2020.

As for other large‐scale datasets, CRISPR screen results can be difficult for researchers to access and interrogate. Complete screen datasets are usually contained in supplemental files and/or stand‐alone resources with inconsistent formats, or presented as raw sgRNA scores that require knowledge of statistical analysis methods to extract meaningful information at the gene or protein level. Rather than re‐analyzing data from the raw sgRNA sequence data, BioGRID curators re‐format and collate each individual screen to improve accessibility in a manner consistent with the published data. ORCS thus captures original screen results exactly as scored with the analytical method used by the authors, including quantitative score values, confidence values, and significance thresholds used to identify hits, when available. In most instances, full genome‐wide datasets are captured in ORCS but in cases where authors report only screen hit lists, these are recorded as published. To allow comparison between disparate screens, ORCS also generates a ranked hit list for each screen based on quantitative scores. This approach of providing original screen scores allows flexibility in the types of screens that can be curated and ensures ORCS is fully compatible with the published literature.

**FIGURE 3:**
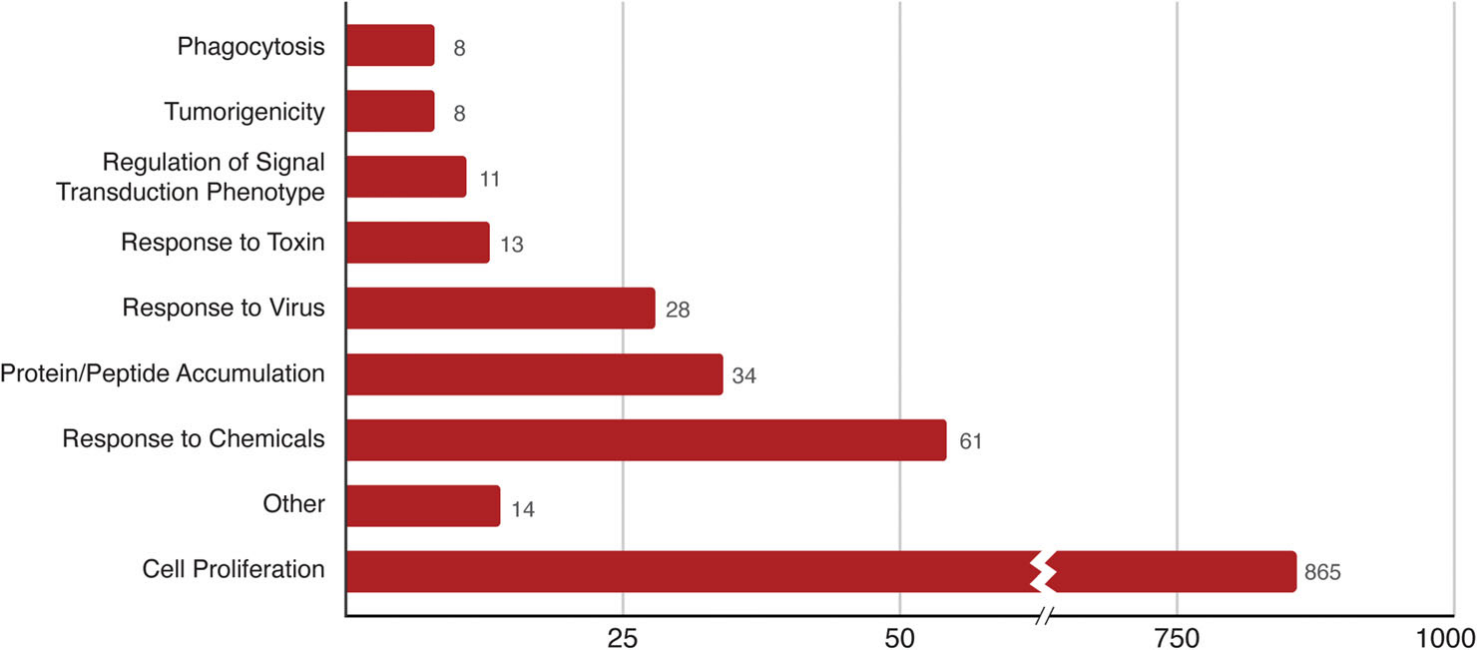
Phenotypes represented in BioGRID‐ORCS dataset. Distribution of screen phenotypes annotated in BioGRID‐ORCS release 1.1.6

**FIGURE 4:**
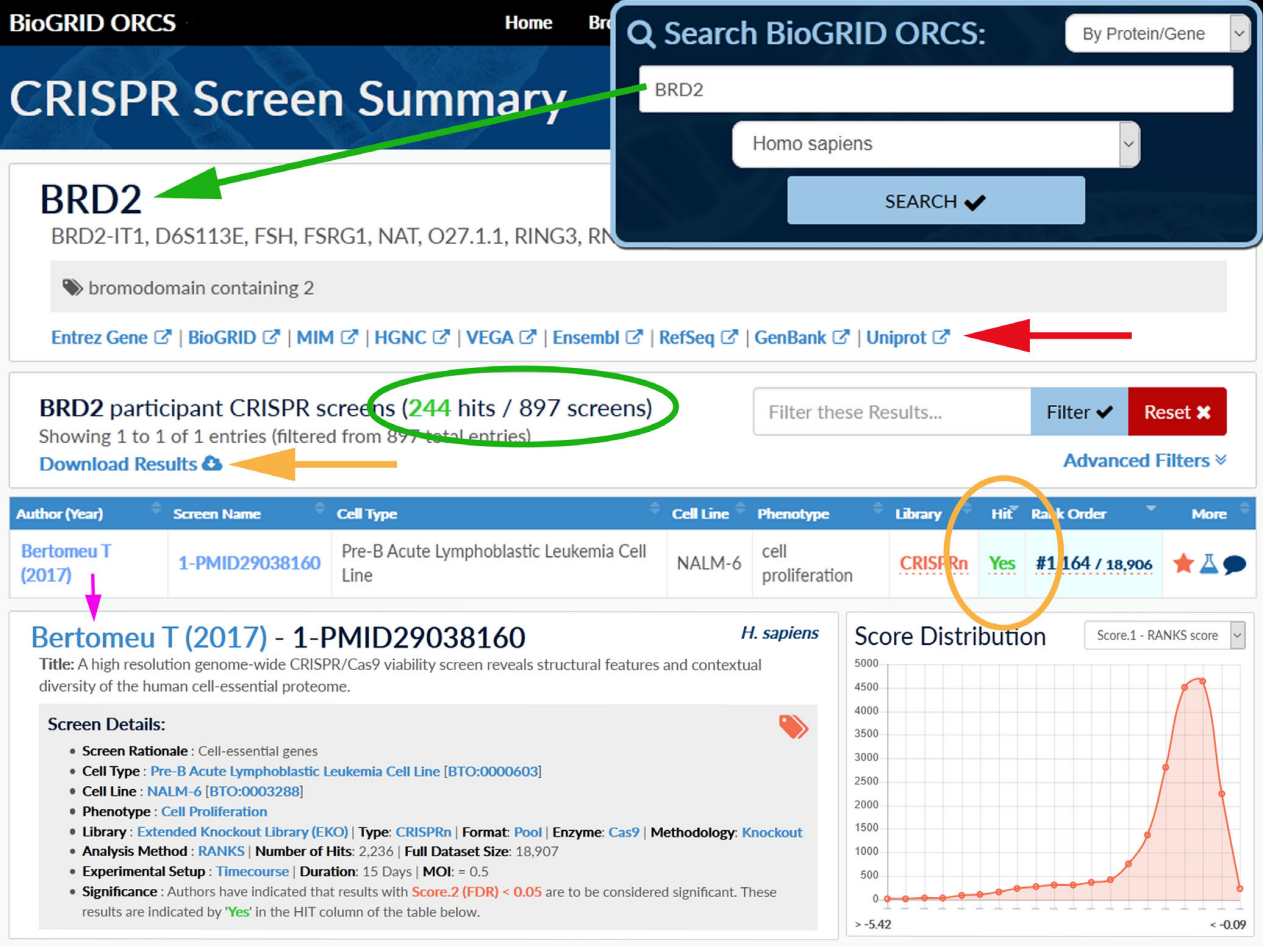
BioGRID‐ORCS screen summary page. Datasets can be browsed by screens or by searching for a gene of interest, as shown here for BRD2. Selecting the species of interest takes the user to a screen results page that lists all screens in which the gene has been tested (large green arrow). Screens in which the gene is designated a hit are listed first and hit rank order in each screen is indicated (orange circle). Overall statistics for the gene are also indicated (green circle). The screen metadata and screen score distribution are displayed by clicking the author link (pink arrow). The full screen dataset can be retrieved by clicking the download button (orange arrow). Linkouts to other resources are also provided (red arrow)

All CRISPR screens are based on phenotypic selection, and hence the scored phenotype is a critical parameter in describing any CRISPR screen. Typically, cell proliferation is the selected phenotype, which can reflect either decreased or increased fitness of the sgRNAs that correspond to any given gene (Figure 3). Screens may be designed to either select specifically for decreased fitness (i.e., negative selection) or increased fitness (i.e., positive selection) or both. Selection of a library pool by outgrowth of a cell line will usually yield only clones that cause cells to grow more poorly and are sometime referred to as essentiality, viability, fitness, or dropout screens. sgRNAs that are depleted in such screens correspond to genes required for optimal cell proliferation. Conversely, screens that enrich for sgRNA‐generated indels that cause resistance to a particular drug,^53^, ^54^, ^55^ toxin,^57^ pathogen,^58,59^ or adverse environmental condition^60^ are termed positive selection screens (Figure 3). Such screens identify genes that mediate the adverse effects of the particular condition, and can provide valuable insight into mechanism of drug action^54–56^ or pathogenesis.^57–59^ Selection at an intermediate drug concentration or level of stress can yield indels that either sensitize or confer resistance to the selection in the same screen.^55,61^ Hit thresholds at one or both sides of the frequency distribution are reported in ORCS whenever defined by the authors. The same principles can be applied to more complex phenotypic readouts, for example, uptake of magnetic particles to evaluate the genetic requirements for phagocytosis.^62^ More sophisticated phenotypic screens represent a growing proportion of the CRISPR screen data in the literature, and will undoubtedly be extended to many different phenotypes, including whole organism contexts.^63^ Arrayed CRISPR libraries coupled with high content image analysis will allow even more complex phenotypes to be interrogated.^64^ To help users interpret complex screens, a screen rationale that succinctly describes the purpose and design of the screen is included in the screen summary (Figure 4).

Users can access the information in ORCS in a variety of formats. Under the browse tab, users can view all screens performed in a particular cell line or by exposure to a drug of interest, or by using the search bar, users can retrieve all screen results for a particular gene/protein, as illustrated for BRD2 (Figure 4). By selecting an organism‐specific ortholog, the user is directed to a CRISPR screen summary page that displays a list of all relevant screens. At the top of this page is general information including links to Uniprot, Entrez Gene and BioGRID and below these links, the number of screens in which the gene is a hit is indicated. In the example shown for BRD2, almost all hits correspond to negative selection screens performed in cancer cell lines, indicating that knock‐out of BRD2 is detrimental in many cancer cell lines and supporting the case for BRD2 as a candidate drug target.^65–67^ Users can interrogate each screen for experimental metadata and ranked hits lists with associated numeric scores. These features allow users to deduce gene/protein function within the context of multiple CRISPR screens.

The flexible nature of the ORCS curation strategy enables curators to quickly incorporate new screen formats. In the context of the COVID‐19 pandemic, BioGRID has prioritized CRISPR screens against SARS‐CoV‐2 and other viruses, which have been carried out in infected primate and human cell line models. To date, BioGRID curation for whole virus screens has covered Ebola, Dengue, HIV, West Nile, Hepatitis C, Influenza A, and SARS‐CoV‐2, among others. Notably, recent preprint reports have described genome‐wide CRISPR screens in the Vero‐E6 African green monkey cell line^68^ and the human HuH‐7.5 liver cancer cell line^69^ subjected to SARS‐CoV‐2 virus infection. As one of these screens was undertaken in the Vero‐E6 cell line, new African green monkey gene annotation was loaded into BioGRID to allow screen curation. CRISPR screens have identified known host cell genes required for viral entry such as ACE2 and heparan sulfate biosynthetic pathway genes, as well as novel host cell dependencies for viral replication including the SWI/SNF chromatin remodeling complex, the TGF‐β signaling pathway, phosphatidylinositol metabolism and endosome maturation, among other processes.^68,69^. These host cell genetic requirements reveal candidate new drug targets for interdiction of the SARS‐CoV‐2 infection cycle.

## 8 SUMMARY AND OUTLOOK

BioGRID will continue to maintain existing data collections and to improve curation throughput by text‐mining approaches. New themed curation projects underway include Alzheimer's disease,^70^ as well as new viral, bacterial and protozoan pathogens, all of which will be supported by dedicated themed project pages. Chemical‐protein interaction datasets will be expanded by de novo curation and key partnerships with chemical and drug databases. BioGRID‐ORCS will capture new CRISPR screen data based on both conventional and more elaborate phenotypic readouts, as well as new CRISPR gene editing systems and libraries. To accommodate new projects and features, the BioGRID is currently being completely rebuilt based on a microservices architecture, which will allow more efficient updates and API interfaces. BioGRID interaction data and CRISPR screen data will continue to be openly disseminated through NCBI, UniProt and other meta‐databases, and we will continue to collaborate on interaction curation and dissemination with the major MODs and their unified resource, the Genome Alliance (www.alliancegenome.org).^71^ All data in BioGRID and ORCS will be maintained with regular updates and made freely available without restriction. BioGRID curation projects will thus continue to serve both the academic biomedical research community and the commercial drug discovery sector.

## AUTHOR CONTRIBUTIONS

**Rose Oughtred:** Conceptualization; data curation; methodology; supervision; writing‐original draft; writing‐review and editing. **Jennifer Rust:** Conceptualization; data curation; methodology; writing‐original draft; writing‐review and editing. **Christie Chang:** Conceptualization; data curation; methodology; writing‐original draft; writing‐review and editing. **Bobby‐Joe Breitkreutz:** Conceptualization; methodology; resources; software; visualization; writing‐original draft; writing‐review and editing. **Chris Stark:** Conceptualization; methodology; resources; software; visualization; writing‐original draft; writing‐review and editing. **Andrew Willems:** Conceptualization; data curation; methodology; writing‐review and editing. **Lorrie Boucher:** Conceptualization; data curation; methodology; supervision; writing‐review and editing. **Genie Leung:** Data curation. **Nadine Kolas:** Data curation. **Frederick Zhang:** Data curation. **Sonam Dolma:** Data curation. **Jasmin Coulombe‐Huntington:** Methodology. **Andrew Chatr‐aryamontri:** Conceptualization; data curation; methodology; writing‐review and editing. **Kara Dolinski:** Conceptualization; funding acquisition; project administration; supervision; writing‐original draft; writing‐review and editing. **Mike Tyers:** Conceptualization; funding acquisition; project administration; supervision; writing‐original draft; writing‐review and editing.

## CONFLICT OF INTEREST

The authors have no conflict of interest to declare.
